# Effects of Undernutrition and Obesity on Functional and Nutritional Outcomes After Ischaemic Stroke: A Hospital‐Based Study

**DOI:** 10.1111/jhn.70240

**Published:** 2026-03-30

**Authors:** Daniela Figueiredo Corrêa Pereira, Karen Barros Parron Fernandes, Andreo Fernando Aguiar, Sergio Marques Borghi, Kamila Grandolfi, Juliano Casonatto

**Affiliations:** ^1^ Research Group in Physiology and Physical Activity University Pitágoras UNOPAR Anhanguera Londrina Paraná Brazil; ^2^ Irmandade Santa Casa de Londrina – ISCAL Londrina Paraná Brazil; ^3^ Department of Medicine Pontifical Catholic University Londrina Paraná Brazil; ^4^ Research Laboratory in Muscular System and Physical Exercise University Pitágoras UNOPAR Anhanguera Londrina Paraná Brazil; ^5^ Center for Research in Biological and Health Sciences University Pitágoras UNOPAR Anhanguera Londrina Paraná Brazil

**Keywords:** disability, nutritional assessment, obesity paradox, stroke outcomes, undernutrition

## Abstract

**Purpose:**

The impact of nutritional status on post‐stroke outcomes remains controversial, particularly regarding the opposing effects of undernutrition and obesity on recovery. This study investigated how these nutritional conditions influence functional and nutritional outcomes among hospitalized ischaemic stroke survivors.

**Methods:**

A retrospective study analyzed 160 adults with radiologically confirmed ischaemic stroke admitted to a Brazilian tertiary hospital. Nutritional status was classified by WHO BMI criteria (undernutrition: BMI < 18.5 kg/m^2^; obesity: BMI ≥ 30 kg/m^2^). Outcomes included discharge/death, length of stay, symptoms/disability (mRS), neurological deficit, food intake, bedridden, respiratory support and enteral nutrition use. Logistic regression models were mutually adjusted for obesity or undernutrition status, depending on the outcome analyzed, to estimate their independent effects.

**Results:**

Undernutrition (*n* = 36) showed strong associations with disability (adjusted OR 6.64, 95% CI 2.19–20.14), symptomatic presentation (aOR 2.84, 1.16–6.94) and reduced food intake (aOR 5.69, 2.28–14.21). Obesity (*n* = 30) was independently linked to higher disability risk after adjustment (aOR 3.32, 1.02–10.80). Estimated marginal probabilities (vs. normal/overweight BMI reference group) revealed that undernourished patients had 47.7% disability risk (vs. 12.1% in nourished) and obese patients 39.2% (vs. 16.2% in non‐obese).

**Conclusions:**

Undernutrition and obesity independently predict worse functional recovery and nutritional outcomes post‐stroke, with undernutrition additionally compromising food intake. The so‐called obesity paradox was not sustained after mutual adjustment. Findings underscore the importance of: (1) early nutritional screening, (2) intensified support for undernourished patients and (3) tailored rehabilitation strategies for obese individuals.

## Summary

- Undernutrition increases post‐stroke disability risk.
- Obesity independently raises disability risk.
- Obesity paradox disappears when controlling for undernutrition.
- Early nutritional screening crucial for stroke recovery.

## 1 Introduction

Stroke remains a leading cause of mortality and long‐term disability worldwide, with ischaemic stroke accounting for ~65% of all cases [1]. Post‐stroke outcomes are influenced by multiple factors, including age, stroke severity and comorbidities [2]. However, the role of nutritional status—particularly undernutrition and obesity—in shaping stroke recovery remains a critical yet underexplored area of research.

Undernutrition, characterized by insufficient nutrient intake and low body mass index (BMI), is a well‐documented risk factor for poor clinical outcomes in acute illness [3]. In stroke patients, undernutrition has been associated with increased infection rates, prolonged hospitalization and higher mortality [4, 5]. Conversely, obesity, defined by excessive adiposity (BMI ≥ 30 kg/m^2^), presents a paradoxical scenario. While obesity is a well‐established risk factor for stroke incidence, some studies suggest it may confer a protective effect in post‐stroke recovery—a phenomenon termed the ‘obesity paradox’ [6, 7]. However, the mechanisms underlying these associations remain unclear, and conflicting evidence highlights the need for further investigation.

Existing studies on nutritional status and stroke outcomes have primarily focused on either undernutrition or obesity in isolation, often neglecting their potential interactions. Recent evidence, including a 2023 meta‐analysis evaluating the Controlling Nutritional Status (CONUT) score in more than 16,000 stroke patients, demonstrated that higher CONUT scores—indicating greater risk of malnutrition—were independently associated with mortality, major disability and infection during hospitalization [8]. These findings reinforce that malnutrition is a key prognostic factor in stroke, but also illustrate that most analyses assess nutritional deficits without simultaneously addressing obesity‐related influences, thereby overlooking possible overlapping mechanisms. Additionally, many analyses fail to account for key functional and neurological outcomes, such as disability severity, mobility status and nutritional interventions during hospitalization.

Although BMI has recognized limitations—particularly its inability to distinguish between lean and fat mass, which may be relevant in older or post‐stroke populations—it remains the most widely used, standardized, and feasible indicator for classifying nutritional status in hospital‐based and epidemiological contexts. Its use facilitates direct comparison with existing literature and enables complementary interpretation.

A comprehensive assessment of how both undernutrition and obesity independently and jointly influence post‐stroke recovery could inform targeted nutritional interventions and improve patient prognoses. Accordingly, this study aimed to investigate the associations between undernutrition, obesity, and clinical outcomes following ischaemic stroke in a hospital setting. We hypothesized that undernutrition would be associated with worse functional and nutritional outcomes, whereas obesity might demonstrate a complex relationship with both adverse and protective effects. By clarifying these associations, our findings seek to support evidence‐based nutritional management strategies and optimize rehabilitation and long‐term recovery in stroke survivors.

## 2 Methods

This study analyzed electronic medical records of ischaemic stroke survivors admitted between January and December 2021 to a tertiary hospital in Londrina, Paraná, Brazil, serving a population of ~600,000 inhabitants. The study protocol received ethical approval from the Institutional Committee on Ethics and Research Involving Human Beings (No. 4.903.486/2021) in accordance with Resolution No. 466/12 of the Brazilian National Health Council and the Helsinki Declaration.

### 2.1 Participants

**Figure 1:**
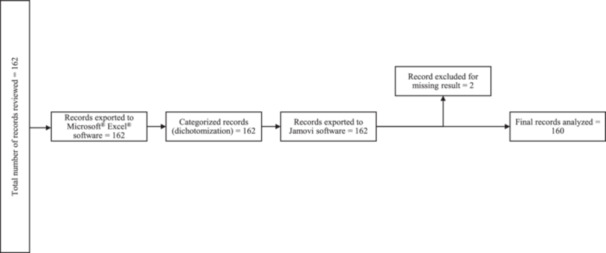
Data flowchart.

A total of 160 adult patients (> 18 years), both sexes, with radiologically confirmed ischaemic stroke who underwent complete nutritional assessment within 72 h of admission were included in the final analysis, while those with haemorrhagic stroke, incomplete data or unconfirmed stroke diagnosis were excluded, as detailed in Figure 1. Individuals with haemorrhagic stroke were excluded to maintain sample homogeneity and reduce clinical heterogeneity, as these conditions differ substantially in aetiology, prognosis and treatment approaches—including nutritional and rehabilitation management. Moreover, haemorrhagic stroke represented a small proportion of admissions during the study period, limiting the feasibility of robust subgroup analyses.

### 2.2 Independent Variables

Nutritional status served as the primary independent variable, with undernutrition classified according to World Health Organization (WHO) criteria as either BMI < 18.5 kg/m^2^ [9]. Obesity was defined as BMI ≥ 30 kg/m^2^ [9]. Body mass and height measurements were obtained by trained clinicians using calibrated equipment during the initial 72‐h admission window.

### 2.3 Dependent Variables

The study examined multiple dependent variables related to hospitalization outcomes, nutritional status and functional recovery. Length of hospital stay was calculated as continuous days from admission to discharge or death and subsequently dichotomized at > 7 days based on the 75th percentile distribution. Clinical outcomes were simply categorized as either discharge (to home or rehabilitation facility) or in‐hospital death.

Nutritional outcomes included food intake, defined as the quantitative estimation of dietary consumption rather than swallowing efficiency. Food intake was assessed using the Pictorial Dietary Assessment Tool (PDAT), a validated instrument developed by Budiningsari et al. [10], which enables healthcare staff to visually estimate the proportion of meals consumed based on standardized food photographs. In our study, the hospital nutrition team applied the PDAT during the first 72 h of admission, collecting data on the patient's habitual dietary intake over the preceding 15 days. The PDAT evaluates macronutrient and energy intake with strong agreement to the food‐weighing method (*r* = 0.84–0.95), providing a simple and valid indicator of hospital food consumption. For analytical purposes, food intake was dichotomized as ‘maintenance’ or ‘reduction’ relative to the patient's usual intake pattern.

Functional assessments incorporated mobility status, where patients unable to sit independently were classified as bedridden, while those with normal or partial gait capacity were considered non‐bedridden.

Patients who required non‐invasive mechanical ventilation were classified. The dichotomization of this variable was ‘yes’ or ‘no’. No patient required invasive mechanical ventilation. Enteral nutrition was characterized by the need for the administration of nutrients directly into the gastrointestinal tract through a tube inserted through the nose, mouth, or abdomen, which reaches the stomach or intestines. The dichotomization of this variable was ‘yes’ or ‘no’.

Neurological evaluation utilized the National Institutes of Health Stroke Scale (NIHSS) [11], with scores > 10 (representing the top quartile) indicating high neurological deficit. The Modified Rankin Scale (mRS) [12] provided measures of both symptoms (asymptomatic for mRS = 0 vs. symptomatic for mRS ≥ 1) and disability (independent for mRS 0–1 vs*.* dependent/impaired for mRS 2–5). Baseline measurements for NIHSS, mRS and nutritional status were obtained within 48 h of admission, with daily monitoring of nutritional and mobility parameters, while final outcome assessments were conducted within 48 h preceding discharge or death to capture the most recent clinical status. The comprehensive data set was extracted from standardized electronic medical records maintained by the hospital's integrated healthcare system.

### 2.4 Statistical Analysis

The statistical analysis employed both descriptive and inferential methods to examine associations between nutritional status (undernutrition and obesity) and clinical outcomes in ischaemic stroke patients. All analyses were conducted using jamovi software (version 2.5.6). Continuous variables are presented as means ± standard deviation, while categorical variables are expressed as frequencies and percentages.

For bivariate analyses examining undernutrition status, obese individuals were systematically excluded from the non‐undernourished reference group to enable precise comparisons between undernourished and adequately nourished (non‐obese, non‐undernourished) participants. Conversely, when analyzing obesity associations, undernourished participants were excluded from the non‐obese reference group. This methodological approach ensured mutually exclusive comparison groups for each nutritional status analysis. These analyses were cross‐sectional, comparing baseline nutritional status against a single measurement of each outcome variable (primarily the assessment closest to discharge). Therefore, between‐group comparisons of categorical variables were performed using chi‐square (*χ* ^2^) tests, with Fisher's exact test substituted when expected cell counts fell below five, as the assumptions of independence between groups were met. Continuous variables were compared using independent samples *t*‐tests.

Logistic regression models were developed to evaluate independent associations between nutritional status and key clinical outcomes, including disability (mRS 2–5), symptomatic presentation (mRS ≥ 1), reduced food intake and prolonged hospitalization (> 7 days). All variables demonstrating associations at *p* < 0.20 in bivariate analyses were considered for inclusion in regression models. Adjusted analyses incorporated obesity status as a covariate in models examining undernutrition outcomes, and reciprocally included undernutrition status when analyzing obesity outcomes. Model adequacy was verified using the Hosmer–Lemeshow goodness‐of‐fit test, while multicollinearity was assessed through variance inflation factors (VIF < 5 considered acceptable).

**Figure 2:**
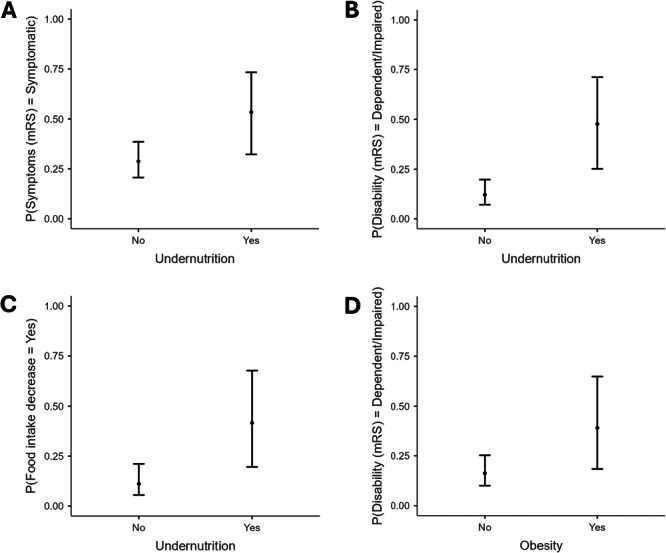
Marginal probabilities (with 95% confidence intervals) of symptomatic presentation, disability and reduced food intake according to undernutrition and obesity status. (A) Symptomatic presentation probability by undernutrition status (marginal estimates with 95% CI: Undernourished = 53.4% [32.3%–73.4%] vs. Normal = 28.8% [20.6%–38.6%]). (B) Disability probability by undernutrition status (marginal estimates with 95% CI: Undernourished = 47.7% [25.2%–71.1%] vs. Normal = 12.1% [7.1%–19.8%]). (C) Food intake reduction probability by undernutrition status (marginal estimates with 95% CI: Undernourished = 41.6% [19.6%–67.6%] vs. Normal = 11.1% [5.5%–21.1%]). (D) Disability probability by obesity status (marginal estimates with 95% CI: Obese = 39.2% [18.4%–64.9%] vs. Non‐obese = 16.2% [10.0%–25.3%]).

Estimated marginal means (EMM) with 95% confidence intervals, derived from adjusted logistic regression models, were graphically presented (Figure 2) to illustrate significant associations. These EMMs represent predicted probabilities of key outcomes across nutritional status groups, with covariates held at their mean values. Statistical significance was defined as *p* ≤ 0.05 for all analyses.

## 3 Results

The general characteristics of the sample are presented in Table 1. The study included 160 participants, comprising 85 males (53.1%) and 75 females (46.9%). The mean age was 68.0 ± 12.1 years for males and 70.8 ± 13.9 years for females, with no statistically significant difference between genders (*t* = −1.398, *p* = 0.164).

**Table 1:** General characteristics of the sample.

	*N*	Range	Minimum	Maximum	Mean	SD	*t*	*p*
*Numerical variables*								
Age (years)								
Male	85	58	38	96	68.0	12.1	−1.398	0.164
Female	75	60	39	99	70.8	13.9		
Length of stay (days)								
Male	85	28	1	28	7.0	5.1	1.535	0.127
Female	75	20	2	22	5.9	3.8		
Neurological deficit (NIHSS score)								
Male	85	17	0	17	6.04	5.00	−1.778	0.077
Female	75	23	0	23	7.51	5.46		

	*N*	No symptoms	No significant	Slight	Moderate	Moderately severe	Severe	
*Categorical variable*								
Functional status (Modified Rankin Scale)—Disability								
Male	85	65 (40.6%)	12 (7.5%)	1 (0.6%)	6 (3.8%)	0 (0.0%)	1 (0.6%)	
Female	75	50 (31.3%)	11 (6.9%)	7 (4.4%)	5 (3.1%)	2 (1.3%)	0 (0.0%)	

Abbreviation: SD = standard deviation.

Regarding hospitalization duration, males had a slightly longer mean length of stay (7.0 ± 5.1 days) compared to females (5.9 ± 3.8 days), though this difference was not statistically significant (*t* = 1.535, *p* = 0.127). Neurological deficit, assessed by the NIHSS score, was 6.04 ± 5.00 in males and 7.51 ± 5.46 in females, showing a trend towards higher disability in females but without reaching statistical significance (*t* = −1.778, *p* = 0.077).

Functional status, evaluated using the mRS, indicated that most participants had no symptoms (40.6% males vs. 31.3% females) or no significant disability (7.5% males vs. 6.9% females). A higher proportion of females exhibited slight (4.4% vs. 0.6%) and moderately severe disability (1.3% vs. 0%), though severe disability was rare in both groups.

Undernutrition demonstrated significant associations with adverse functional and nutritional outcomes (Table 2). Patients with undernutrition had markedly higher rates of disability (33% dependent/impaired vs. 7%, *p* < 0.001) and symptomatic presentation (44% vs. 22%, *p* = 0.019). They were also more likely to experience reduced food intake (56% vs. 18%, *p* < 0.001). Notably, trends suggesting potential associations were observed for enteral nutrition use (33% vs. 18%, *p* = 0.084) and neurological deficit severity (33% high severity vs. 25%, *p* = 0.077), meeting the predefined threshold (*p* < 0.2) for inclusion in subsequent regression analyses.

**Table 2:** Association of undernutrition and obesity with clinical outcomes, disability and nutritional interventions in hospitalized patients.

	Undernutrition				Obesity			
	No % (*N*)	Yes % (*N*)	*χ* ^2^	*p*	No % (*N*)	Yes % (*N*)	*χ* ^2^	*p*
LOS								
≤ 7 days	69% (69)	81% (22)	1.631	0.202^a^	69% (69)	67% (20)	0.058	0.809^a^
> 7 days	31% (31)	19% (5)			31% (31)	33% (10)		
Clinical outcome								
Discharge	87% (87)	93% (25)	0.638	0.424^b^	87% (87)	83% (25)	0.260	0.610^a^
Death	13% (13)	7% (2)			13% (13)	17% (5)		
Symptoms (mRS)								
Asymptomatic	78% (78)	56% (15)	5.463	**0.019** a	78% (78)	63% (19)	2.621	0.105^a^
Symptomatic	22% (22)	44% (12)			22% (22)	37% (11)		
Disability (mRS)								
Independent	93% (93)	67% (18)	13.389	**< 0.001** a	93% (93)	80% (24)	4.333	**0.037** a
D/I	7% (7)	33% (9)			7% (7)	20% (6)		
ND (NIHSS)								
Low	75% (75)	67% (18)	0.753	0.077^a^	75% (75)	87% (26)	1.812	0.178^b^
High	25% (25)	33% (9)			25% (25)	13% (4)		
Food intake decrease								
No	82% (82)	44% (12)	15.591	**< 0.001** a	82% (82)	93% (28)	2.277	0.131^b^
Yes	18% (18)	56% (15)			18% (18)	7% (2)		
Bedridden								
No	56% (56)	41% (11)	1.986	0.159^a^	56% (56)	69% (20)	1.561	0.211^a^
Yes	44% (44)	59% (16)			44% (44)	31% (9)		
NIMV								
No	91% (91)	93% (25)	0.068	0.794^b^	91% (91)	97% (29)	1.044	0.307^b^
Yes	9% (9)	7% (2)			9% (9)	3% (1)		
Enteral nutrition								
No	82% (82)	67% (18)	2.986	0.084^a^	82% (82)	93% (28)	2.277	0.131^b^
Yes	18% (18)	33% (9)			18% (18)	7% (2)		

*Note:* Bold values indicate statistically significant at *p* < 0.05.

Abbreviations: D/I = dependent/impaired, LOS = length of stay, mRS = Modified Rankin Scale, ND = neurological deficit, NIHSS = National Institutes of Health Stroke Scale, NIMV = non‐invasive mechanical ventilation.

^a^ *χ* ^2^ test.

^b^ Fisher's exact test.

In contrast, obesity was significantly associated with higher disability rates (20% dependent/impaired vs. 7%, *p* = 0.037). Non‐significant trends were noted for milder neurological deficits (13% high severity vs. 25%, *p* = 0.178) and reduced enteral nutrition need (7% vs. 18%, *p* = 0.131), which also qualified for regression analysis.

No significant associations were found between nutritional status and length of stay (undernutrition: *p* = 0.202; obesity: *p* = 0.809) or mortality (undernutrition: *p* = 0.424; obesity: *p* = 0.610). These outcomes did not meet the threshold for further regression analysis.

In unadjusted analyses (Table 3), undernutrition emerged as the strongest predictors of disability (mRS: OR 4.50, 95% CI 1.68–12.04, *p* = 0.003) and reduced food intake (OR 6.87, 95% CI 2.81–16.84, *p* < 0.001). Symptomatic status (mRS: OR 2.35, 95% CI 0.99–5.53, *p* = 0.050) and enteral nutrition use (OR 2.75, 95% CI 1.08–6.98, *p* = 0.033) also showed significant associations. A trend was observed for bedridden status (OR 2.09, 95% CI 0.90–4.85, *p* = 0.088), while neurological deficit was non‐significant (*p* = 0.227).

**Table 3:** Logistic regression analysis of factors associated with undernutrition and obesity.

		Unadjusted model			Adjusted by obesity		
Variable	Reference	OR (95% CI)	*β* (SE)	*p*	OR (95% CI)	*β* (SE)	*p*
Undernutrition							
Symptoms (mRS)	Asymptomatic	2.35 (0.99–5.53)	0.85 (0.43)	**0.050**	2.84 (1.16–6.94)	1.04 (0.46)	**0.022**
Disability (mRS)	Independent	4.50 (1.68–12.04)	1.50 (0.50)	**0.003**	6.64 (2.19–20.14)	1.89 (0.57)	**< 0.001**
ND (NIHSS)	Low	1.74 (0.71–4.28)	0.55 (0.46)	0.227	1.50 (0.60–3.76)	0.40 (0.47)	0.387
Food intake decrease	No	6.87 (2.81–16.84)	1.93 (0.46)	**< 0.001**	5.69 (2.28–14.21)	1.74 (0.47)	**< 0.001**
Bedridden	No	2.09 (0.90–4.85)	0.73 (0.43)	0.088	1.85 (0.78–4.39)	0.61 (0.44)	0.162
Enteral nutrition	No	2.75 (1.08–6.98)	1.01 (0.47)	**0.033**	2.28 (0.88–5.88)	0.82 (0.48)	0.089
Obesity							
Symptoms (mRS)	Asymptomatic	1.58 (0.68–3.66)	0.46 (0.43)	0.283	2.05 (0.85–4.95)	0.72 (0.45)	0.109
Disability (mRS)	Independent	1.73 (0.61–4.89)	0.55 (0.53)	0.298	3.32 (1.02–10.80)	1.20 (0.60)	**0.046**
ND (NIHSS)	Low	0.42 (0.14–1.29)	−0.87 (0.57)	0.131	0.46 (0.15–1.45)	−0.77 (0.58)	0.186
Food intake decrease	No	0.20 (0.04–0.90)	−1.59 (0.76)	**0.036**	0.32 (0.07–1.49)	−1.12 (0.78)	0.148
Enteral nutrition	No	0.26 (0.06–1.18)	−1.33 (0.76)	0.082	0.32 (0.07–1.49)	−1.12 (0.78)	0.148

*Note:* Hosmer–Lemeshow goodness‐of‐fit test, with *p* = 1.000 for all models. Bold values indicate statistically significant at *p* < 0.05.

Abbreviations: 95% CI = 95% confidence interval, mRS = Modified Rankin Scale, ND = Neurological deficit, NIHSS = National Institutes of Health Stroke Scale, OR = odds ratio, ß (SE) = unstandardized log‐odds coefficient (estimate) with standard error.

After adjustment for obesity, the association between undernutrition and disability strengthened (aOR 6.64, 95% CI 2.19–20.14, *p* < 0.001), as did symptomatic presentation (aOR 2.84, 95% CI 1.16–6.94, *p* = 0.022). Reduced food intake remained a robust predictor (aOR 5.69, 95% CI 2.28–14.21, *p* < 0.001), though its effect size slightly attenuated. Enteral nutrition retained a borderline association (aOR 2.28, 95% CI 0.88–5.88, *p* = 0.089), while bedridden status and neurological deficit became non‐significant (*p* > 0.15).

The logistic regression analysis of factors associated with obesity revealed distinct patterns in both unadjusted and adjusted models (Table 3). In unadjusted analyses, obesity demonstrated significant protective effects against reduced food intake (OR 0.20, 95% CI 0.04–0.90, *p* = 0.036) and showed a trend towards lower enteral nutrition use (OR 0.26, 95% CI 0.06–1.18, *p* = 0.082). A potentially important but non‐significant association emerged for milder neurological deficits (OR 0.42, 95% CI 0.14–1.29, *p* = 0.131). Neither symptomatic status (OR 1.58, *p* = 0.283) nor disability (OR 1.73, *p* = 0.298) showed significant associations in these crude models.

After adjustment for undernutrition status, several notable changes occurred in the obesity associations. Most strikingly, disability became significantly associated with obesity (aOR 3.32, 95% CI 1.02–10.80, *p* = 0.046), suggesting this relationship was masked in unadjusted analyses. The previously observed protective effects against food intake reduction (aOR 0.32, *p* = 0.148) and enteral nutrition use (aOR 0.32, *p* = 0.148) both lost significance when accounting for undernutrition. The trend towards milder neurological deficits persisted but remained non‐significant (aOR 0.46, *p* = 0.186), while symptomatic status showed a new marginal association (aOR 2.05, *p* = 0.109) after adjustment.

The EMM analysis revealed significant differences in outcome probabilities for the key adjusted model associations. For undernutrition outcomes (Figure 2A), symptomatic presentation probability nearly doubled from 28.8% (95% CI 20.6%–38.6%) in non‐undernourished patients to 53.4% (95% CI 32.3%–73.4%) in undernourished cases. Disability probabilities (Figure 2B) showed an even more pronounced disparity, with undernourished patients having a 47.7% probability (95% CI 25.2%–71.1%) of dependency compared to just 12.1% (95% CI 7.1%–19.8%) in adequately nourished patients. The probability of reduced food intake (Figure 2C) was 3.7 times higher in the undernourished group (41.6%, 95% CI 19.6%–67.6%) versus controls (11.1%, 95% CI 5.5%–21.1%).

For obesity‐related outcomes (Figure 2D), the probability of disability was 2.4 times greater in obese patients (39.2%, 95% CI 18.4%–64.9%) compared to non‐obese patients (16.2%, 95% CI 10.0%–25.3%).

## 4 Discussion

This study provides compelling evidence that nutritional status—both undernutrition and obesity—significantly influences functional and nutritional outcomes following ischaemic stroke. Our findings demonstrate that undernutrition is strongly associated with increased disability, symptomatic presentation and reduced food intake, while obesity shows a more complex relationship with post‐stroke disability, potentially masking some associations until accounting for undernutrition status. These results highlight the critical role of nutritional assessment in stroke management and suggest that tailored interventions may be necessary for patients at opposite extremes of the nutritional spectrum.

### 4.1 Undernutrition as a Predictor of Poor Stroke Outcomes

The most striking finding of this study was the robust association between undernutrition and adverse functional outcomes. Patients with undernutrition had a 4.5‐fold higher unadjusted odds of disability (mRS 2–5) and a 6.6‐fold increased risk after adjusting for obesity, reinforcing undernutrition as an independent predictor of poor recovery. These results align with previous studies linking malnutrition to muscle wasting [13], impaired immune [14] and metabolic function [15]. Notably, undernourished individuals also exhibited significantly reduced food intake (56% vs. 18%), suggesting that inadequate nutritional support during hospitalization may exacerbate functional decline.

The doubled probability of symptomatic presentation (mRS ≥ 1) in undernourished patients (53.4% vs. 28.8%) reinforces the clinical importance of early nutritional assessment. While existing studies have examined undernutrition's association with complications such as infections [16] and prolonged hospitalization [4], our findings specifically highlight its strong relationship with disability and loss of functional independence—outcomes that directly impact rehabilitation potential and long‐term quality of life after stroke. This distinction underscores the need to expand nutritional evaluations beyond traditional morbidity metrics to include functional recovery measures.

### 4.2 The Dual Role of Obesity in Stroke Recovery

Contrary to the ‘obesity paradox’ observed in some studies [6, 7], our adjusted analyses revealed that obesity independently increased the odds of disability (aOR 3.32, *p* = 0.046). This association was only apparent after controlling for undernutrition, suggesting that obesity's effects may be obscured in unadjusted models. One possible explanation is that obese patients, despite having greater energy reserves, may experience metabolic dysfunction [17], chronic inflammation [18] or reduced mobility [19], all of which could impair stroke recovery.

Interestingly, obesity initially appeared protective against reduced food intake (OR 0.20, *p* = 0.036) and enteral nutrition need (OR 0.26, *p* = 0.082) in unadjusted analyses. However, these associations lost significance after adjustment, implying that confounding by undernutrition status may have influenced earlier observations. This finding challenges the notion that obesity universally confers post‐stroke benefits and instead supports a more nuanced interpretation—where obesity may mitigate acute nutritional deficits but ultimately worsens functional outcomes.

### 4.3 Clinical and Nutritional Implications

The lack of association between nutritional status and mortality or hospital stay duration contrasts with some prior studies [5, 8] but may reflect differences in care protocols or sample characteristics. However, the strong links between undernutrition and disability, food intake, and enteral nutrition use highlight critical clinical implications. These findings support the implementation of routine nutritional screening within 72 h of stroke admission to promptly identify high‐risk patients. For undernourished individuals, comprehensive and multidisciplinary nutritional support—encompassing individualized dietary planning, regular reassessment of intake and needs, close collaboration with dietitians and early initiation of enteral feeding when oral intake is insufficient—may help mitigate functional decline and promote recovery. Similarly, obese stroke survivors may benefit from tailored rehabilitation strategies that address mobility limitations.

### 4.4 Discharge Practices and the Deprioritization of Nutritional Care

Our findings showed that patients identified as undernourished within the first 72 h of hospitalization experienced greater disability and poorer outcomes by discharge. Although nutritional status was evaluated early during admission, it is possible that not all undernourished patients remained malnourished at the time of discharge. Nonetheless, the strong association between early undernutrition and unfavourable outcomes underscores the clinical relevance of nutritional care from the beginning of hospitalization.

In many tertiary hospitals, discharge decisions are primarily driven by neurological stability and overall medical recovery, while nutritional recovery often receives less attention in discharge criteria. This imbalance may lead to premature transitions to outpatient or rehabilitation settings before nutritional needs are fully addressed. Strengthening discharge protocols to include nutritional parameters—such as improvements in intake or risk score reduction—and ensuring the involvement of dietitians in multidisciplinary care planning could help bridge this gap. Such practices would promote continuity of nutritional rehabilitation and potentially improve long‐term functional outcomes after stroke [20].

### 4.5 Limitations and Future Directions

This study has several limitations, including its retrospective design and reliance on a single centre, which may affect generalizability. The absence of body composition measures, such as muscle mass or fat distribution, also limits the ability to fully assess obesity's metabolic impact.

Another important limitation of this study is the absence of detailed data on acute treatment pathways, such as intravenous thrombolysis, mechanical thrombectomy or other reperfusion interventions. These procedures are known to significantly influence both mortality and functional recovery after ischaemic stroke and could act as confounding factors in the observed associations. Consequently, our findings should be interpreted as associations rather than causal relationships, recognizing that part of the effect attributed to nutritional status may reflect differences in treatment access or eligibility. Future studies incorporating detailed clinical intervention data are warranted to further clarify these relationships.

Future research should also prioritize prospective, multicentre studies examining long‐term functional outcomes beyond hospitalization and assess the efficacy of targeted nutritional interventions (e.g., protein supplementation, micronutrient optimization) in reducing disability risk. Additionally, investigations into the underlying mechanisms—such as inflammation, metabolic dysregulation or muscle catabolism—linking nutritional status to neurological recovery could further elucidate causal pathways and support the development of individualized nutritional strategies for stroke survivors.

Additionally, medication use during hospitalization and at discharge—including antiplatelet agents, anticoagulants, antihypertensives and statins—was not consistently recorded in the data set and, therefore, could not be included in the analyses. Because these drugs can meaningfully influence post‐stroke recovery trajectories, complication rates and survival, residual confounding related to pharmacological treatment cannot be excluded [21]. Future studies with comprehensive medication data are warranted to better isolate the independent effects of nutritional status on clinical outcomes.

## 5 Conclusion

In this sample of ischaemic stroke survivors, undernutrition was a strong predictor of disability and reduced food intake, while obesity independently increased disability risk after accounting for confounding factors. These findings challenge simplistic interpretations of the obesity paradox and highlight the need for nutritionally stratified care pathways in stroke management. Early identification and intervention for both undernourished and obese patients may improve functional recovery and reduce long‐term dependency.

## Author Contributions

**Daniela Figueiredo Corrêa Pereira:** conceptualization, data curation, formal analysis, investigation, methodology, writing – original draft, writing – review and editing. **Karen Barros Parron Fernandes:** methodology, supervision, validation, writing – review and editing. **Andreo Fernando Aguiar:** formal analysis, methodology, visualization, writing – review and editing. **Sergio Marques Borghi:** data curation, resources, software, validation. **Kamila Grandolfi:** investigation, project administration, data collection, writing – review and editing. **Juliano Casonatto:** conceptualization, funding acquisition, supervision, project administration, writing – review and editing.

## Funding

The authors have nothing to report.

## Ethics Statement

The study protocol received ethical approval from the Institutional Committee on Ethics and Research Involving Human Beings (No. 4.903.486/2021) in accordance with Resolution No. 466/12 of the Brazilian National Health Council and the Helsinki Declaration.

## Conflicts of Interest

The authors declare no conflicts of interest.

## Data Availability

Data will be available based on the request from the corresponding author.
